# Sclerokeratoplasty for the early management of acquired anterior staphyloma

**Published:** 2019

**Authors:** Guilherme Malta Pio, Pio Frederico Malta, Rodrigues Leonardo Pereira, Rezende Aline Vilani da Silva, Cordeiro Frederico de Miranda

**Affiliations:** * Department of Ophthalmology, Instituto de Olhos Ciências Médicas, Belo Horizonte/ MG, Brazil; ** Department of Surgical Retina, Hospital São Geraldo - HC/ UFMG, Belo Horizonte/ MG, Brazil; *** Department of Cornea, Hospital São Geraldo - HC/ UFMG, Belo Horizonte/MG, Brazil

**Keywords:** corneal injuries, corneal ulcer, anterior staphyloma, sclerokeratoplasty

## Abstract

**Objective:** To report a case of acquired anterior staphyloma after trauma and its first surgical management.

**Methods:** This is a case report of a 17-year-old man who had a history of trauma by insect on the right eye, without a previous history of eye conditions, and evolved with local pain and low visual acuity. The ophthalmological exam showed light perception visual acuity in right eye and 1,0 in left eye, anterior staphyloma and impossibility to blink. The first surgical procedure proposed was sclerokeratoplasty and the second one an optical transplantation but, after step one, the patient did not return to the service and missed the follow-up.

**Results:** Sclerokeratoplasty was proposed once the posterior segment and the crystalline were preserved in topical position. The anterior tumor was excised in free-cut and corneal-scleral graft sutured in single points with 10-0 mononylon. Gatifloxacin 0.3% with Prednisolone Acetate 1%, Epitezan® and Atropine 1% were prescribed immediately postoperative. After 60 postoperative days, he maintained the use of Dexamethasone 0.1% and Atropine 1% and the patient had visual acuity of perception of hand movement in the affected eye.

**Conclusion:** Few treatment options are alternatives to evisceration. In this case report, the sclerokeratoplasty was the chosen technique for the initial management. The second step was not possible due to loss of follow-up. Despite the uncomplicated procedure, we need greater compliance by the patient to conclude the treatment.

## Introduction

The anterior staphyloma, less frequent than the posterior, is a protrusion of the uveal tissue due to a corneal and/ or scleral defect [**1**-**3**]. It can be classified as acquired (as in cases of ocular trauma, corneal ulcers, and vitamin A deficiency) or congenital (such as the one occurring in the Peters anomaly) [**2**].

In general, they are associated with perforated corneal ulcers of fungal origin and result from situations in which treatment was not instituted or did not present responses [**1**-**3**]. Regardless of the cause, it is correlated with a low visual acuity and has poor prognosis [**4**].

Many cases culminate with evisceration. Therefore, emergency measures must be taken to avoid the spread of microorganisms, leading to endophthalmitis and compromise the internal structures of the eyeball [**4**,**5**].

The few treatment options, alternatives to mutilation, include penetrating keratoplasty, sclerokeratoplasty, mushroom graft and dural patch [**3**,**5**-**7**]. Due to the technical difficulty of treatment, the therapeutic options should be evaluated in a systematic and criterious way, in order to reduce intra and postoperative complications.

## Case report

A 17-year-old male patient, previously healthy was admitted to the ophthalmological emergency with complaint of progressive right eye (RE) tumor, associated with mild local pain and low visual acuity. He reported the onset of the condition 5 months before, after eye trauma with insect when riding a motorcycle. He denied previous eye medical history.

**Fig. 1,2:**
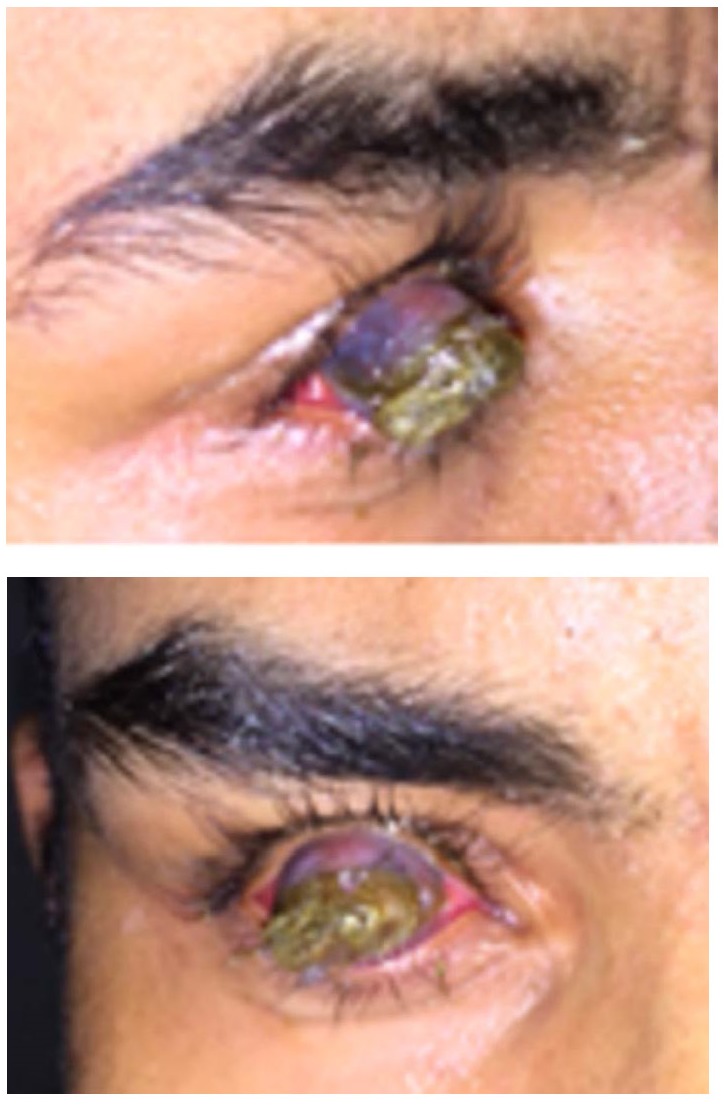
Patient at admission, with presence of anterior lesion in the right eye. Left eye without changes

The following were observed on examination: RE with visual acuity of light perception, corneal-scleral region opaque with anterior staphyloma approximately two centimeters long, necrotic tissue and exit of thick secretion in the outermost region, associated with adhered pilification, making it impossible to blink. Examination of left eye without changes and 20/ 20 visual acuity without correction (**Fig. 1,2**).

**Fig. 3A,B:**
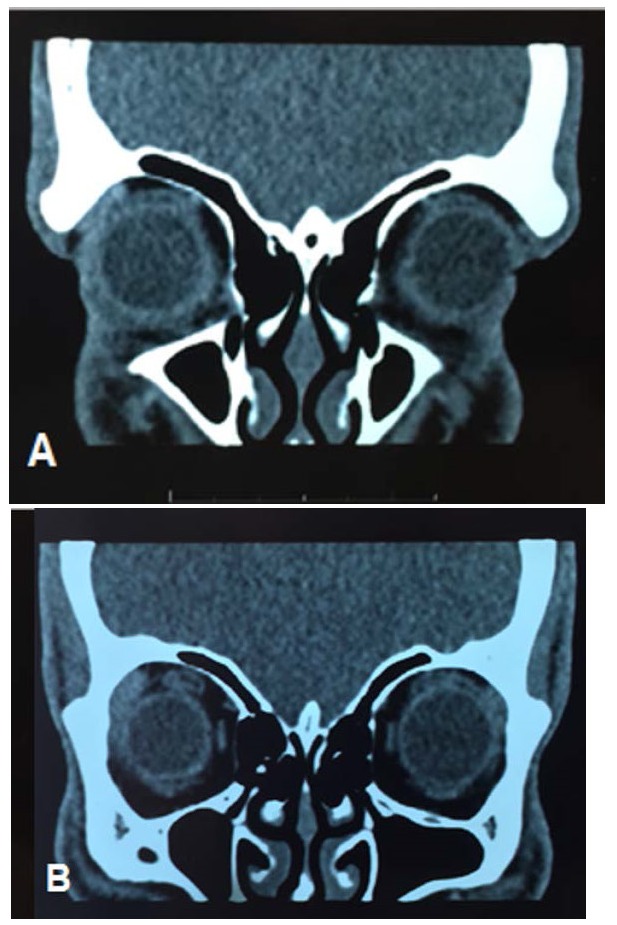
Computed tomography of orbit showing preservation of structures external to the eyeball

Ultrasound B of the affected eye without changes in the posterior segment, similar to the contralateral eye and topical crystalline. Computed tomography of orbits showing a lesion restricted to the eyeball (**Fig. 3A,3B**).

**Fig. 4,5:**
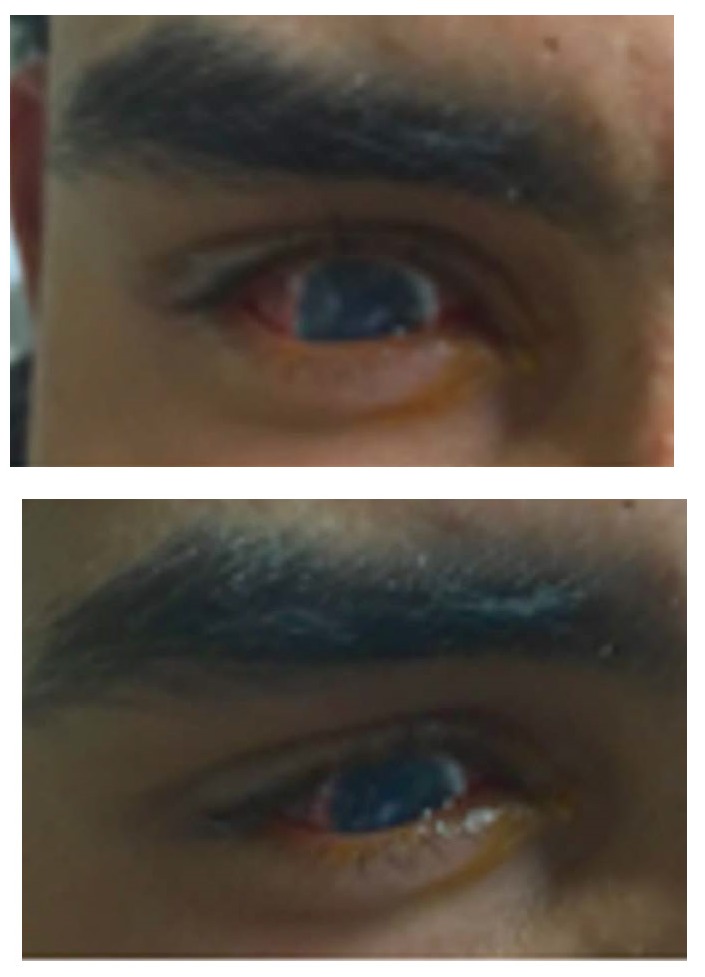
Patient after 60 days of sclerokeratoplasty

**Fig. 6:**
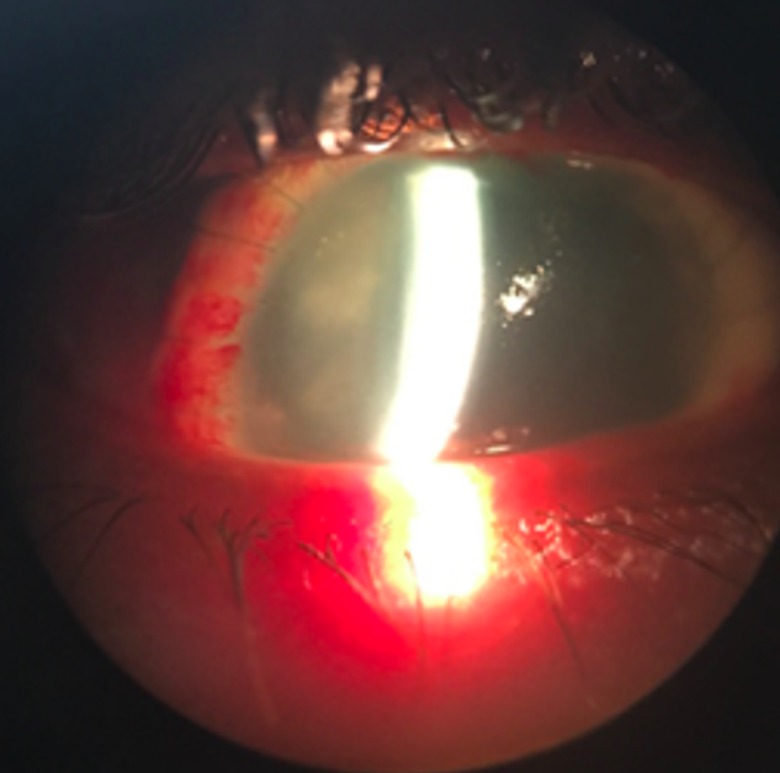
60 days after sclerokeratoplasty - vision by the slit lamp

After 20 days of patient admission and results of imaging tests, sclerokeratoplasty was proposed. Due to the size of the lesion, the superior portion of the staphyloma was explored and manual trephination by limbal peritomy with a scalpel blade 11 was performed. Trephine was used to open 7.50 mm upper staphyloma’s side and its resection until limbal corneal portion, were done. Corneal-scleral graft, measuring 8.00 mm, was a suture with 10-0 mononylon in separate single stitches. Immediate postoperative association of Gatifloxacin 0.3% with Prednisolone Acetate 1% 4/ 4 hours eye drops, Epitezan® TID ointment and Atropine 1% BID eye drops were prescribed. The patient evolved on first day postoperative with intense conjunctival hyperemia, in addition to superior scleral tapering, but there was progressive improvement of the conjunctival condition. The graft button was already opacified, neovasous growth throughout its length, without areas of de-epithelialization and anterior chamber remained formed, although it was shallow. Clinical compensation was initially opted for because it was a tectonic transplant. After 60 postoperative days, still using Dexamethasone 0.1% TID eye drops and Atropine 1% BID eye drops, examination with visual acuity of perception of hand movement in the affected eye was done. Therefore, there was a satisfactory result, with good esthetics of the ocular surface reconstruction and positive response to the patient’s expectations (**Fig. 4**-**6**).

The follow-up of the case provided an optical transplant after a critical period of adaptation of the corneal-scleral graft, but there was loss of follow-up and the patient did not return to the service.

## Discussion

The acquired anterior staphyloma occurs when there is corneal perforation and subsequent adhesion of the uveal tissue to the lesion [**1**-**3**,**7**]. Due to the vascularization of the iris, a pseudocornea is formed, being unable to withstand intraocular pressure and, consequently, results in progressive protrusion [**1**,**4**,**6**].

The treatment of anterior staphyloma usually involves evisceration of the affected eye, and sclerokeratoplasty is a therapeutic alternative in order to restore the eyeball and prepare for a second intervention [**5**,**6**,**8**].

Graft surgery involves greater complexity, largely due to loss of limbic support and consequent intraoperative iris diaphragm protrusion [**3**,**6**,**7**]. The patient described was submitted to sclerokeratoplasty in order to avoid evisceration and with the objective of future refractive keratoplasty. In this particular case, therapeutic, tectonic, and cosmetic requirements were met in order to perform the corneoscleral transplantation. Although the procedure occurred without complications, the possible long-term complications were not known or complementary procedures were performed in the patient, since there was loss of follow-up in the service.

**Declaration of interest**

None. All authors declare no conflict of interest in this work. All authors contributed equally to this work.
